# Civilization and Decay of the Teeth

**Published:** 1882-01

**Authors:** 


### CIVILIZATION AND DECAY OF THE TEETH.

The current progress of caries with civilization is admitted on all hands. The jaw of the Zulu or aboriginal Australian presents no traces of this malady ; the teeth may be worn down, even to the edge of the gum, by constant grinding of the unground maize, but no chemical action of the fluids of the mouth is visible, the ravages of fungous growths are not present. The degeneration and early destruction of the wisdom teeth in European skulls, as compared with those of uncivilized tribes or the anthropoid Apes, is also an admitted fact. Another curious observation has just been made. Dr. Beddoe and Mr. Tuckett state that the average British head is undergoing a steady diminution in size, amounting to three-quarters of an inch during the last twenty-five years, as shown by the circumferential measurement of hatters. These facts all seem to show that civilization is tending to a deterioration in the structure and build of the human frame, and nothing leads to this conclusion more surely than a consideration of the details of the processes by which the teeth are destroyed.—*Journ. Brit. Dent. Asso'n.*

